# Functions of Flavonoids in Plant–Nematode Interactions

**DOI:** 10.3390/plants7040085

**Published:** 2018-10-15

**Authors:** Sabrina Chin, Carolyn A. Behm, Ulrike Mathesius

**Affiliations:** 1 Division of Plant Sciences, Research School of Biology, Australian National University, Canberra 2601, Australia; Sabrina.Chin@anu.edu.au; 2 Division of Biomedical Science and Biochemistry, Research School of Biology, Australian National University, Canberra 2601, Australia; Carolyn.Behm@anu.edu.au

**Keywords:** auxin, chemotaxis, flavonoid, gall, motility, nematode, syncytia

## Abstract

Most land plants can become infected by plant parasitic nematodes in the field. Plant parasitic nematodes can be free-living or endoparasitic, and they usually infect plant roots. Most damaging are endoparasites, which form feeding sites inside plant roots that damage the root system and redirect nutrients towards the parasite. This process involves developmental changes to the root in parallel with the induction of defense responses. Plant flavonoids are secondary metabolites that have roles in both root development and plant defense responses against a range of microorganisms. Here, we review our current knowledge of the roles of flavonoids in the interactions between plants and plant parasitic nematodes. Flavonoids are induced during nematode infection in plant roots, and more highly so in resistant compared with susceptible plant cultivars, but many of their functions remain unclear. Flavonoids have been shown to alter feeding site development to some extent, but so far have not been found to be essential for root–parasite interactions. However, they likely contribute to chemotactic attraction or repulsion of nematodes towards or away from roots and might help in the general plant defense against nematodes. Certain flavonoids have also been associated with functions in nematode reproduction, although the mechanism remains unknown. Much remains to be examined in this area, especially under field conditions.

## 1. Introduction to Plant Parasitic Nematodes

Nematodes are small roundworms with a bilateral symmetry and unsegmented bodies [1]. Whilst most nematodes are free-living, ~7% (>4100 species) of the characterized nematodes belong to the plant-parasitic nematode (PPN) group [2,3,4]. PPNs are agricultural pests that cause significant crop damage and crop loss, estimated at up to $US 125 billion globally per annum [4,5,6,7]. This is due to the diversion of host nutrients to PPNs and interference with transport processes, as well as physical damage caused during feeding or migration, which can also result in secondary infections [8,9].

PPNs are classified into three orders—the Triplonchida, Dorylaimida, and Tylenchida—with the majority of agriculturally damaging nematodes belonging to the last order [10]. These PPNs have evolved a highly specialised feeding structure, termed the stylet, to feed on plant tissues, and often display complex life-stages to suit their environment [11,12,13]. The tylenchids are classified based on their trophic niche, either as aerial nematodes or root parasitic nematodes [10].

## 2. Plant–Nematode Interactions

**Figure 1:**
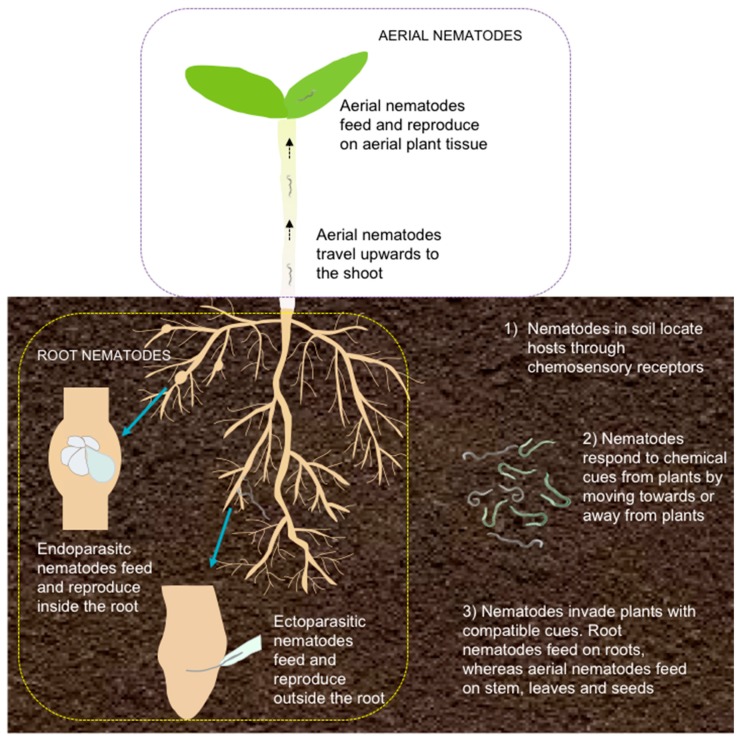
Summary of interactions between plant hosts and plant-parasitic nematodes. Plant hosts are infected by both root and aerial nematodes. Their interaction starts in the soil with the perception of host cues by the nematode, followed by attraction or repulsion towards or away from the host. Root nematodes include ecto- and endoparasites, with endoparasites causing the greatest damage due to their induction of a complex feeding site, in which they reproduce.

Plant–nematode interactions often begin in the soil (Figure 1), where the PPNs perceive various host cues using chemosensing, mechanosensing, thermosensing, redox potential sensing, humidity sensing, osmotic sensing, and electrosensing [14,15,16]. It is thought that PPNs possess similar neuroanatomy and neurobiology as the free-living nematode model, *Caenorhabditis elegans*, and that taxis towards a source of plant cues relies mainly on chemosensation, although these processes are poorly understood in PPNs [14,17].

Chemosensation in PPNs is strongly linked to their host range, with PPNs with narrow host ranges thought to have sensitive chemosensation, such as the potato cyst nematode, *Globodera pallida* and *G. rostochiensis*, and the soybean cyst nematode, *Heterodera glycines*, as they respond very strongly to specific chemicals in the root exudates by hatching and moving towards the chemical [18,19]. In contrast, PPNs with a broad host range, such as the root-knot nematodes, *Meloidogyne* spp., also rely on non-specific abiotic cues, namely low pH and CO_2_ gradients [20,21]. These signals are concurrently analyzed by the chemoreceptors in the anterior receptors, the amphids, and in some PPNs, the posterior receptors, the phasmids, to determine the orientation of the PPN [15,19]. In the event of a positive response, the PPN orients itself towards the cue and begins its migration towards the source (Figure 1). If the PPN does not find a compatible cue within its pre-parasitic life cycle (i.e., egg and juvenile stages), it will reduce its metabolism, either by undergoing a quiescence process—e.g., the pre-parasitic juvenile nematode ceases movement until stimulated—or it will enter a diapause process such as delaying egg hatching [22,23].

The next interaction occurs at the root interface, whereby root nematodes penetrate the root tissue or remain external to the root, whereas aerial nematodes continue to migrate upwards to the stem (Figure 1). Next, the PPNs commence feeding and mature, and finally start to reproduce inside or outside the host. Aerial nematodes can feed on the bulb, stem, and foliage, whereas root nematodes feed exclusively on the root [10,24]. Root PPNs deploy different parasitic strategies, being (1) either migratory or sedentary during feeding, and (2) being either endoparasitic or ectoparasitic during feeding and reproduction [10,25,26]. The most damaging PPNs belong to the sedentary endoparasitic group, the root knot nematodes (*Meloidogyne* species) and cyst nematodes (*Globodera* and *Heterodera* species), followed by the migratory endoparasites, the root lesion nematodes (*Pratylenchus* species) and the burrowing nematodes (*Radopholus* species) [9,27]. The success of root sedentary endoparasites can be attributed to the sophisticated exploitation of many different plant response pathways to alter plant defense responses and to induce long-term feeding sites, and to the difficulty in diagnosing infections due to below ground symptoms [24,27]. Overall, there has been limited success in controlling PPNs via chemicals, biological control, or creating effective plant resistance [28,29,30]. Delivering nematode resistance has included attempts at overexpression of specific genes, e.g., proteinase inhibitors, or expression of RNAi constructs targeting nematode-specific genes in transgenic plants [28]. In addition, several resistance genes effective against parasitic nematodes have been cloned, many of which resemble genes conferring resistance to other pathogens. For example, the *Mi*, *Hiro A*, *Gpa2*, and *Gro1-4* genes belong to the class of NBS-LRR genes and confer resistance to a number of endoparasitic nematodes [31]. Other resistance genes, like *Rhg1* and *Rhg4* from soybean encode proteins with extracellular LRR motifs, while others, like *Hsp1^pro1^* do not show similarity to other known genes [31]. So far, there has been limited success in transferring these resistance genes to heterologous species. Resistance responses conferred by R genes include activation of a number of defense responses, including hypersensitive response, to limit the spread of the pathogen. Flavonoids are one class of plant metabolites that have been associated with enhanced resistance to pathogens, including nematodes.

## 3. Flavonoids in Plants

Flavonoids constitute a large class of secondary carbon-based metabolites present in all land plants. More than 10,000 different types of flavonoids have been described from a variety of plant species. Flavonoids are a class of phenylpropanoids derived from the shikimate and acetate pathways through the activity of a cytosolic multienzyme complex anchored to the endoplasmic reticulum and typically contain a diphenylpropane backbone (C3-C6-C3), which forms the basis of flavonoid subgroup classification [32]. There are several flavonoid subgroups based on their structural properties, including the chalcones, flavones, flavonols, flavandiols, anthocyanins, condensed tannins, aurones, isoflavonoids, and pterocarpans [33,34,35]. Flavonoids within the subgroups are extensively modified through secondary modifications of the backbone, for example by hydroxylation, glycosylation, methylation, malonylation, prenylation, acylation, dehydration, and polymerization [36]. The functions of individual flavonoids are strongly affected by their structure and include roles in plant development via the control of auxin transport, flower pigmentation, as antioxidants (ROS scavengers), as defense compounds, chemoattractants, signals for plant–microbe interactions (notably nodulation), male fertility in some species and help in nutrient mining [35]. Flavonoids are actively exuded into the rhizosphere, likely using ABC transporters and multidrug and toxic compound extrusion (MATE) transporters in both aglycone and glycosidic forms [37,38,39]. Small amounts of flavonoids also diffuse into the soil during root cap sloughing [40]. Most studies on flavonoid exudation have measured flavonoid concentrations from the nano- to micromolar ranges in growth medium under semi-sterile laboratory conditions [39]. Hence, their functions, bioavailability, mobility, concentrations, and gradients in real soil situations with rhizosphere microorganisms are still poorly understood. Here, we focus on the known roles of flavonoids as defense compounds and as developmental regulators during feeding site formation.

## 4. Flavonoids as Defense Compounds against Nematodes

**Figure 2:**
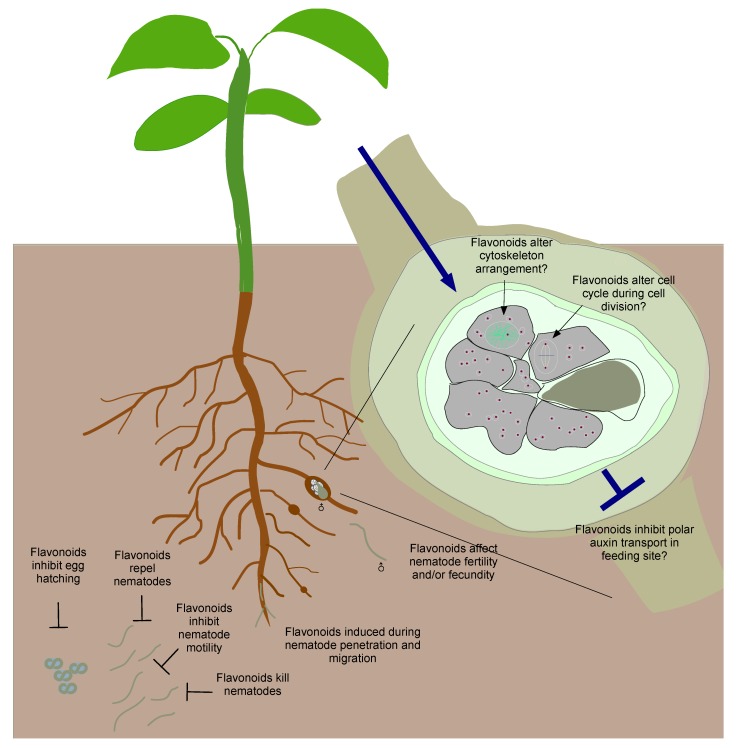
Flavonoids play multiple roles during plant–nematode interactions. The example shown here is for root-knot nematodes. Flavonoids in the rhizosphere can have effects on the pre-parasitic stages by inhibiting egg hatching, repelling hatched nematodes, inhibiting their movement and by killing them. Synthesis of flavonoids is induced when the nematodes penetrate and migrate inside the root; they can act as defense compounds or signals for defense via cross-talk with other defense/stress pathways. Flavonoids can affect adult stages of nematodes by altering their fertility and/or fecundity. The role(s) of flavonoids in feeding site development is less understood. They are postulated to be involved in the inhibition of auxin transport, cell cycle regulation, and cell cytoskeleton rearrangement.

A PPN will first encounter flavonoids in the soil when it is locating its host (Figure 1). This can occur whilst the PPN is in the egg or juvenile stage. For PPNs in the egg stages, flavonoids can inhibit egg hatching (Figure 2), as shown in a study by Wuyts and colleagues [41], in which kaempferol inhibited *Radopholus similis* egg hatching. As for juvenile PPNs, flavonoids can: (1) induce quiescence by slowing down their movement, resulting in periods of reversible inactivity; (2) modify their migration towards the roots by repelling them; and (3) kill them (Figure 2). For example, the flavonols kaempferol, quercetin, and myricetin repelled and slowed *M. incognita* juveniles at micromolar concentrations [41]. Patuletin, patulitrin, quercetin, and rutin were shown to kill the juveniles of *H. zeae* at various concentrations and durations of exposure [42]. Flavonoid effects on PPNs are also species-specific. Using similar concentrations of flavonols, kaempferol, quercetin, and myricetin repelled *M. incognita* and *R. similis* juveniles, but not *Pratylenchus penetrans,* whereas the flavonols inhibited the motility of *M. incognita* juveniles but not *R. similis* and *P. pratylenchus* juveniles [41]. Interestingly, in *C. elegans* exposure of young adults (L4 stage) to 100µM flavonols, particularly kaempferol in liquid and plate media, prolonged their lifespan through effects on an ageing-associated gene, the transcription factor DAF-16, and by reducing mitochondrial reactive oxygen species (ROS) [43,44,45]. The differences in flavonoid effects in different nematode species is likely due to the differences in chemosensory receptors, flavonoid receptor binding affinities, cell signaling cascade, and solute permeability across the cuticle in different species, although this has not been studied yet. Furthermore, not much is known about the existence or functions of putative flavonoid receptors in any PPN.

Once the PPN has reached the plant, it inflicts mechanical damage to the plant tissue to penetrate and/or to feed on the tissue. This is followed by the production and release of defense compounds (i.e., phytoalexins and phytoanticipins) to respond to PPN attack (Figure 2). Although some flavonoids such as (*E*)-chalcone, patuletin, and rutin killed pre-parasitic stages of cyst nematodes [42,46], their accumulation, concentrations, and activity in planta is unclarified. In addition, numerous studies have found increased flavonoid gene expression and flavonoid accumulation at infections sites of both endo- and ectoparasitic PPNs, and induction of flavonoids has repeatedly been found to be higher in resistant compared with susceptible host cultivars (summarised in Table 1). Flavonoids that have most commonly been implicated as defense compounds against PPNs mostly belong to the isoflavonoids and pterocarpan classes, (e.g., coumesterol, glyceollin (soybean-specific), formononetin, and medicarpin) as well as the flavonols (e.g., kaempferol and quercetin) (Table 1). Some studies have also shown that flavonoid glycosides are likely involved in defense, such as medicarpin glucoside malonate and formononetin glucoside malonate [47].

The plant host and the PPN itself can manipulate the flavonoid biosynthesis pathway during PPN pathogenesis directly or indirectly. One study suggested that yellow-coloured cyst nematodes, *G. pallida* and *G. rostochiensis*, modified quercetin and kaempferol into a nematode-unique flavonoid, quercentagetin [48]. Flavonoids are likely taken up by the PPN’s digestive system as the PPN feeds on the cytoplasmic content. In endoparasitic PPNs, flavonoids may also diffuse through the cuticle from nearby plant cells that surround the parasite within the root tissue. Nonetheless, it is not well understood to what extent flavonoids accumulate inside nematodes, how they are processed, or whether or not they play a role in the infection process.

The flavonoid biosynthesis pathway in the plant can be induced by a broad pathogenesis response through jasmonic acid, salicylic acid, ethylene, auxin, and ROS cross-talks, likely triggered when the PPNs cause mechanical damage and wounding during feeding and penetration [49,50]. Flavonoids are also likely to be released from storage in the cytosol, vacuole, endoplasmic reticulum, chloroplast, nucleus, and small vesicles during tissue damage or cell rupture [51]. Flavonoids have been found to be induced in roots of plants only infected by nematodes in the shoot, suggesting that systemic signals may induce flavonoid synthesis in infected plants, but so far it is unknown what these systemic signals are [52]. Some of the flavonoid biosynthesis pathways can be manipulated by the PPN via the secreted enzyme chorismate mutase, which regulates the shikimate pathway and thereby the downstream flavonoid, salicylic acid, auxin, and amino acid biosynthesis pathways in the plant [53]. Chorismate mutase gene(s) or enzymes have been found in juveniles of endoparasitic PPNs such as *M. javanica, M. incognita, M. arenaria, H. glycines, H. schachtii*, and *G. pallida,* in the esophageal glands, and are potentially involved in the induction of their feeding sites, after being secreted into giant cells [54,55,56,57,58,59,60].

The flavonoids that accumulate at PPN feeding sites may affect nematode fertility and fecundity (Figure 2) by limiting egg production or skewing the ratio of males to females, as more females are formed under abundant nutrition and vice versa (e.g., *Meloidogyne* spp. and *Heterodera* spp.) [61]. A study by Jones et al. (2007) found that infection of *transparent testa* (*tt*) mutants of *Arabidopsis,* which are defective in parts of the flavonoid pathway, i.e., *tt4/tt6*, *tt4/tt5*, and *tt6*, resulted in an increased number of female cyst nematodes [62]. In contrast, a similar study by Wuyts et al. [63] using different Arabidopsis flavonoid mutants, i.e., *tt3*, *tt4*, *tt5*, and *tt7*, infected with *M. incognita,* found that the defects in the flavonoid pathway did not affect the number of adult females, egg masses, eggs, or juveniles. A systematic dissection of the effects of specific flavonoid metabolites on fertility in different types of nematodes still remains to be done.

## 5. Flavonoids Play Minor Roles in the Development of Nematode Feeding Sites

Feeding sites are essential to the survival and the establishment of PPN as parasites. The PPNs feed on the cytoplasm of the cells, and sometimes mitochondria and plastids, using their stylet. Feeding sites are usually established by the second-stage juvenile (pre-parasitic stage), with some exceptions in *Naccobus aberrans*, *Tylenchus semipenetrans*, *Rotylenchulus* spp., whereby the adult females induce the feeding sites [83].

PPNs form a variety of feeding sites, which are influenced by their life-style (e.g., migratory vs sedentary), with different degrees of plant cell manipulation. Migratory PPNs such as *Bursaphelenchus*, *Aphelenchoides* spp., and *Pratylenchus* spp. typically do not induce feeding sites, but rather feed off plant material directly, causing physical wounding. Sedentary PPNs with extended feeding duration induce specific and complex feeding sites inside their hosts. These include galls induced by root knot nematodes, which are characterized by multinucleate giant cells, and syncytia induced by the cyst nematodes. Inside the cells of the feeding site, PPNs cause multiple host cell responses to increase the starch, sugar, and amino acid content, and turning the feeding cell into a metabolic sink by increasing transporter and plasmodesmatal networks, increasing cell surface area by cell wall invagination, and altering cell metabolism [84].

Feeding site formation also involves control of the plant cell cycle. Giant cells originate from approximately 3–10 procambium cells within the root endodermis. These cells undergo multiple rounds of endoreduplication and acytokinetic mitosis, resulting in enlarged, multi-nucleated cells with dense cytoplasm and elaborate ingrowths [85,86]. The hypertrophy of giant cells and nematode enlargement causes secondary cell divisions in the surrounding pericycle and cortical cells to accommodate this growth [87]. As a result, galls, or ‘root knots’, are formed on the root. Similar to giant cells, syncytia are also multinucleate but are formed from the protoplast fusion and cell wall dissolution of several adjacent pericycle or procambium cells [88]. As there are no secondary cell divisions, no gall is formed.

Flavonoids may be involved in the regulation of polar auxin transport to enhance auxin accumulation in nematode feeding sites (Figure 2). Some flavonoids inhibit cell-to-cell polar auxin transport and/or the inhibiting auxin efflux transporters, PIN (Pin-formed) and PGP (P-Glycoprotein) [89,90]. In addition, some flavonoids can control auxin content by regulating IAA (indoleacetic acid) oxidase [91]. The initiation and development of both types of feeding sites requires local auxin accumulation and redistribution for cell division, cell differentiation, cell wall loosening and the growth of new vascular tissue [92,93,94]. Studies by Kyndt and colleagues [95] as well as Grunewald and colleagues [96] showed that root knot and cyst nematodes modulate PIN protein localisation to redistribute auxin in feeding sites and neighboring cells. For example, the expression of *PIN2* and *PIN7* was suppressed in giant cells and syncytia, presumably to increase auxin transport into those cells. Furthermore, transcriptomic and proteomic analysis in root knot and cyst nematode-infected roots demonstrated a correlation between flavonoid gene/protein expression with auxin inducible gene/protein expression. For instance, Oliveira and colleagues [79] found the chalcone flavone isomerase and an auxin-induced protein were upregulated in cowpea at four to six days post inoculation with *M. incognita*, whereas Ithal and colleagues [80] found upregulation of several flavonoids in soybean (e.g., chalcone synthase, chalcone isomerase, isoflavone reductase) and *PIN2* transcripts in cyst nematode-infected roots. A study by Hutangura and colleagues [81] observed that the induction of *CHS1* and *CHS2* (chalcone synthase, the first enzyme in flavonoid biosynthesis) in root-knot nematode galls coincided spatially and temporally with increased auxin response, within 120 h of inoculation. These studies suggest that flavonoids could be employed by nematodes early during feeding site development to facilitate auxin accumulation. Nevertheless, there are gaps in the link between flavonoids and auxins in nematode feeding sites, as there has neither been any research demonstrating that in vitro or in planta manipulation of flavonoids resulted in the inhibition of PIN protein function. We also do not know which specific flavonoid metabolites are active in this process. We suggest that flavonols such as kaempferol, quercetin and their glycosides would be likely used by these nematodes for auxin regulation as they have been shown to inhibit polar auxin transport [94,97,98].

Even though flavonoids may be involved in feeding site development, they appeared not to be essential and are unlikely to be involved in feeding site initiation, as Wasson and colleagues [82] showed that root knot nematodes can still initiate galls in flavonoid-deficient roots of *Medicago truncatula*. However, these galls were reported to be smaller, with reduced numbers of dividing pericycle cells, perhaps due to reduced local auxin accumulation in the gall. This suggests that flavonoids may be required to maintain local auxin maxima in feeding sites for long-term maintenance and development. As mentioned above, Wuyts and colleagues [63] also showed that flavonoid deficiency in Arabidopsis did not alter the infection and reproduction capacity of *M. incognita*.

Flavonoids may also be involved in the cell cycle regulation of PPN feeding sites (Figure 2). PPN feeding sites commonly contain enlarged nuclei with higher DNA content compared with other cells [99], a process achieved through endoreduplication in the S-phase of mitosis during cell proliferation [100]. It is presumed that endoreduplication is a strategy used to increase DNA content and gene dosage, thereby increasing cell metabolism and growth in feeding sites [101]. These processes are mostly studied in giant cells and syncytia, in which endoreduplicating cells bypass the transition from G2 to mitosis and remain in repeated transitions between G2-, G1-, and S-phases via the activity of cyclin-dependent kinases and other regulators e.g., CCS52 [102,103]. Flavonoids such as quercetin, genistein, persicogenin, artemetin, luteolin, penduletin, and vitexicarpin inhibit cell cycle progression from G2 to mitosis and induce apoptosis in mammalian models [104]. It is plausible that flavonoids could be used to regulate endoreduplication by PPNs with an additional regulation to prevent apoptosis, but this has not been substantiated in plant models. Furthermore, the cell cycle regulation in giant cells is complicated by the switch between endoreduplication and acytokinetic mitosis, indicating the ability of root-knot nematodes to up and downregulate different sets of cyclin-dependent kinases and potentially specific types of flavonoids.

Giant cells and syncytia undergo extensive cytoskeleton rearrangement for their initiation and development. Root knot nematodes induce partial depolymerisation of actin filaments, particularly in phragmoplasts (resulting in incomplete cytokinesis) in giant cells, whereas cyst nematodes induce complete depolymerisation of actin filaments in syncytia, although it unknown how this occurs [105]. In addition, root knot and cyst nematodes can modify actin transcription. For instance, the actin genes *ACT2* and *ACT7* were upregulated in giant cells and syncytia during early infection [106]. These PPNs may partly achieve this via flavonoids (Figure 2), as Böhl and colleagues [107] discovered that kaempferol, quercetin, fisetin, and genistein could depolymerise actin and inhibit actin transcription in a dose-dependent manner at micromolar concentrations. In contrast, epigallocatechin stimulated actin polymerisation and transcription [107]. So far, flavonoid deficient mutants have not been utilised to study the effects of flavonoids on the cytoskeleton inside feeding sites in planta.

## 6. Summary and Outlook

Flavonoids can play multiple roles during plant–nematode interactions by acting as defense compounds or signals that directly and indirectly affect nematode fitness at different life stages. Several studies have shown effects of flavonoids on the survival of nematode eggs, on the fecundity of nematodes and on the attraction of nematodes towards host roots. However, most of these studies require validation in plants and utilising definitive flavonoid mutants of host plants. There is a general trend that specific flavonoids are induced during plant–nematode interactions, especially in feeding sites. There is also evidence in some interactions that more nematode-resistance plant genotypes accumulate higher concentrations of flavonoids that might act as phytoalexins. However, absence of flavonoids in host plants has been shown not to prevent the formation of feeding sites of sedentary PPN. Therefore, it appears more likely that the roles of flavonoids in plant–nematode interactions are in defense, rather than developmental control. Future research could be directed at identifying mechanisms by which flavonoids directly act on nematode behavior and survival, as well as trying to enhance nematode resistance by engineering host plants with increased content of flavonoids acting as phytoalexins.

## Figures and Tables

**Table 1:** Summary of the involvement of plant flavonoids in plant–nematode interactions. Rows in red indicate studies demonstrating the role of flavonoids in nematode defense responses, whereas rows in blue indicate studies demonstrating the role of flavonoids in nematode feeding sites and rows in purple indicate studies intersecting across both roles.

Name of Enzyme/Gene/Metabolite	Flavonoid Accumulation Site	Suggested Flavonoid Function	Host Studied	Nematode Studied	Reference
Glyceollin	Roots	Glyceollin I and III accumulated more in the resistant cultivar compared with the susceptible cultivar.	Soybean, *Glycine max*	Soybean cyst nematode, *Heterodera glycines*	[64]
	Stele in roots	Glyceollin was associated with the incompatible interaction between the resistant cultivar and *M. incognita*: accumulation was localised in the stele of resistant roots, high concentrations of glyceollin in resistant cultivar and glyceollin inhibited the motility of *M. incognita.*	Soybean, *Glycine max*	Root-knot nematodes, *Meloidogyne incognita*. and *M. javanica*	[65]
	Leaves	Glyceollin accumulated at sufficiently high concentrations at infection sites to result in a localised hypersensitive response. It inhibited nematode motility and respiration as well as plant tissue death via inhibition of mitochondrial electron transport system.	In vitro system	Root-knot nematodes, *Meloidogyne incognita*, and *M. javanica*	[66]
Phaseollin	Hypocotyl and root	Phaseollin found only in *P. penetrans* infected tissue. The survival of *P. penetrans* juveniles incubated for 16 h in 47 µg/mL of phaseollin solution was unaffected.	Common bean, *Phaseolus vulgaris*	Root-lesion nematode, *Pratylenchus penetrans*	[67]
Sakuranetin	Leaf	Present only in resistant cultivars—suggested to be involved in defense	Rice	Stem nematode, *Ditylenchus angustus*	[68]
Formononetin and Formononetin-7-O-glu-coside-6”-O-malonate Medicarpin-3-O-gluco-side-6”-O-malonate Medicarpin Coumesterol glucosides	Roots, meristems, leaves	Isoflavonoid and pterocarpan (conjugate) accumulation correlated with nematode resistance. Medicarpin inhibited *P. penetrans* in a concentration dependent manner.	White clover, *Trifolium repens*	Stem nematode, *Ditylenchus dipsaci*	[47]
			Lucerne, *Medicago sativa*	Stem nematode, *Ditylenchus dipsaci*	[52]
			Lucerne, *Medicago sativa*	Root-lesion nematode, *Pratylenchus penetrans*	[69]
O-methyl-apigenin-C-hexoside-O-deoxyhexoside Apigenin-C-hexoside-O-pentoside Luteolin-C-hexoside-O-pentoside	Roots and shoots during *P. neglectus* and *H. avenae* infection	Flavonoids possibly acted as broad defense compounds—induced in methyl jasmonate and nematode-treated plants. Plants treated with root extracts from methyl jasmonate-induced plants had reduced infection.	Oats, *Avena sativa*	Root lesion nematode, *Pratylenchus neglectus,* Cereal cyst nematode, *Heterodera avenae,* Stem nematode, *Ditylenchus dipsaci*	[70]
Coumesterol Psoralidin	Roots	Coumesterol and psoralidin accumulated in roots and were localised at lesion sites caused by nematodes only in lima bean. Coumesterol significantly inhibited nematode motility at 10–15 µg/mL concentrations.	Lima bean, *Phaseolus lunatus* and snap bean, *P. vulgaris*	Root-lesion nematode, *Pratylenchus scribneri*	[71]
Quercentagetin (hydroxy-flavone) Aurone Chalcone	Adult female extracts	The yellow coloration in *G. rostochiensis and G. pallida* is attributed to flavonoid quercetagetin, which was present in pathotypes with yellow color and absent in paler pathotypes.	N/A	Potato cyst nematodes, *Globodera rostochiensis* and *G. pallida*	[48]
Flavan-3,4-diols Condensed tannins	Roots	Flavan-3,4-diols and condensed tannins accumulated after nematode infection.	Banana, *Musa*	Burrowing nematode, *Radopholus similis*	[72]
Daidzein Genistein Other isoflavonoids	Roots	Daidzein and genistein increased in susceptible Sussex cultivar at two and four days post inoculation. Isoflavonoid production was enhanced in nematode infected plants in susceptible Sussex and resistant Hartwig cultivar at two and three days post inoculation.	Soybean, *Glycine max*	Soybean cyst nematode, *Heterodera glycines*	[73]
Several compounds from the chalcone, flavone, flavanone, isoflavonoid and flavonol pathways.	Purified compounds and plant extracts.	Kaempferol, quercetin and myricetin repelled *R. similis* and *M. incognita* juveniles at 60–84 µg/mL. Luteolin, daidzein and genistein, repelled *R. similis* at 100–142 μg/mL. Kaempferol, quercetin, myricetin, rutin and quercitrin inhibited 13–41% of *M. incognita* juveniles after 48 h of incubation. Naringenin and hesperetin, apigenin, daidzein, and kaempferol reduced egg hatching in *R. similis* up to 21%.	N/A	Burrowing nematode, *Radopholus similis*, root-lesion nematode, *Pratylenchus penetrans* and root-knot nematode, *Meloidogyne incognita*	[41]
Patuletin Patulitrin Quercetin Rutin	Purified compounds and marigold, *Tagetes patula* L. flower extracts	Patuletin killed 100% of nematodes at various dilutions after 72 h, whereas patulitrin killed 10–50% and quercetin killed 70–80% of nematodes. Rutin at 0.5–1% killed all nematodes within 24 h.	N/A	Corn cyst nematode, *Heterodera zeae*	[42]
(*E*)-chalcone	Purified compound	(*E*)-chalcone killed nematodes at 33 μM within 24 h and completely inhibited egg hatching at <10 μM within 15 days.	N/A	Potato cyst nematodes, *Globodera rostochiensis* and *G. pallida*	[46]
Phenylalanine ammonia lyase (PAL)	Roots	Plants grown at 27 °C had optimal PAL activity, which enabled the plants to synthesise phenylpropanoids used in nematode defense, as opposed to 32 °C, which inhibited enzyme activity.	Tomato, *Lycopersi- cum esculentum*	Root-knot nematode, *Meloidogyne incognita*	[74]
Phenylalanine ammonia lyase Chalcone synthase Chalcone isomerase Isoflavone reductase Caffeic acid *O-*methyltransferase 4-coumarate-CoA ligase Cinnamoyl CoA reductase Dihydroflavonol 4-reductase	Roots	Gene expression levels generally induced by nematodes and higher in resistant plants.	Alfalfa, *Medicago sativa*	Root-lesion nematode, *Pratylenchus penetrans*	[69]
			Soybean, *Glycine max*	*Heterodera glycines* and *Meloidogyne incognita*	[75]
			Cowpea. *Vigna unguiculata* L. Walp	*Meloidogyne incognita*	[76]
			Soybean, *Glycine max* genotype PI 88788	Soybean cyst nematode, *Heterodera glycines* population NL1-RHg/HG-type 7	[77]
Flavonoid pathways: *Arabidopsis:* chalcone synthase, chalcone isomerase and flavonoid 3’ hydroxylase, dihydroflavonol 4-reductase Tobacco: Phenylalanine ammonia lyase, anthocyanidins	Roots	*M. incognita* reproduction was significantly higher in tobacco mutant with higher anthocyanidin content. *M. incognita* reproduction in *Arabidopsis tt* mutants and wild-type plants were similar.	Tobacco, *Nicotiana tabacum* and *Arabidopsis thaliana*	Root-knot nematode, *M. incognita*	[63]
Quercetin 7-glucoside and other phenols	Root extracts	Root extracts inhibited nematode motility, reduced nematode egg hatching and reduced gall numbers.	*Lantana camara* L.	Root-knot nematode, *Meloidogyne javanica*	[78]
Chalcone isomerase Auxin-induced protein	Roots	Chalcone isomerase protein as well as an auxin-induced protein were increased at 4, 5, and 6 days post inoculation.	Cowpea. *Vigna unguiculata* L. Walp	Root-knot nematode, *Meloidogyne incognita*	[79]
Chalcone synthase Chalcone flavanone isomerase Isoflavone reductase (putative) Dihydroflavonol 4-reductase Quercetin 3-O-methyltransferase 4-coumarate-CoA ligase *PIN 2*	Root tissue	Upregulation of flavonoid synthesis and *PIN 2* genes in nematode infected roots.	Soybean, *Glycine max* L. Merr. cv. Williams 82	Soybean cyst nematode, *Heterodera glycines* Ichinohe	[80]
Chalcone synthase genes, *CHS1, CHS2, CHS3* Auxin responsive gene, *GH3*	Root tissue	*CHS1::gusA, CHS2::gusA* and *CHS3::gusA* expressions overlapped with *GH3::gusA* expression at 48 h, 72 h and 120 h post inoculation.	White clover, *Trifolium repens* cv. Haifa	Root-knot nematode, *Meloidogyne javanica*	[81]
tt4 (chalcone synthase) mutant, tt5 (chalcone isomerase) mutant tt6 (flavonoid 3’ hydroxylase) mutant	N/A	Flavonoid deficiency in *tt (transparent testa)* mutant lines of single and double *tt4*, *tt5*, and *tt6* did not reduce the number of adult females, with several lines producing more female nematodes.	*Arabidopsis thaliana*	Sugar beet nematode, *Heterodera schachtii*	[62]
Chalcone synthase (silencing by RNA interference)	Root tissue	Flavonoid deficiency did not affect gall numbers. Flavonoid deficient roots had shorter galls and less pericycle cell division compared to roots with flavonoids.	Barrel medic, *Medicago truncatula*	Root-knot nematode, *Meloidogyne javanica*	[82]

## References

[1] Abad P., Williamson V.M. (2010). Plant nematode interaction: A sophisticated dialogue. Adv. Botanical Res..

[2] Bird A.F. (1987). Moulting of parasitic nematodes. Int. J. Parasitol..

[3] Decraemer W., Hunt D.J., Perry R.N., Moens M. (2006). Structure and classification. Plant Nematology.

[4] Nicol J.M., Turner S.J., Coyne D.L., Nijs L.D., Hockland S., Maafi Z.T. (2011). Current nematode threats to world agriculture. Genomics and Molecular Genetics of Plant-Nematode Interactions.

[5] Sasser J.N., Freckman D.W., Veech J.A., Dickson D.W. (1987). A World Perspective on Nematology: The Role of the Society. Vistae on Nematology.

[6] Chitwood D.J. (2003). Research on plant-parasitic nematode biology conducted by the United States Department of Agriculture–Agricultural Research Service. Pest Manag. Sci..

[7] McCarter J.P., Berg R.H., Taylor C.G. (2009). Molecular approaches toward resistance to plant-parasitic nematodes. Cell Biology of Plant Nematode Parasitism.

[8] Perry R.N., Moens M., Jones J., Gheysen G., Fenoll C. (2011). Introduction to plant-parasitic nematodes; Modes of parasitism. Genomics and Molecular Genetics of Plant-Nematode Interactions.

[9] Jones J.T., Haegeman A., Danchin E.G., Gaur H.S., Helder J., Jones M.G., Perry R.N. (2013). Top 10 plant-parasitic nematodes in molecular plant pathology. Mol. Plant Pathol..

[10] Tytgat T., De Meutter J., Gheysen G., Coomans A. (2000). Sedentary endoparasitic nematodes as a model for other plant parasitic nematodes. Nematology.

[11] Grandison G.S. (1977). Relationships of plant-parasitic nematodes and their hosts. New Z. Entomol..

[12] Quitst C.W., Smant G., Helder J. (2015). Evolution of plant parasitism in the phylum Nematoda. Annu. Rev. Phytopathol..

[13] Kikuchi T., Eves-van den Akker S., Jones J.T. (2017). Genome evolution of plant-parasitic nematodes. Annu. Rev. Phytopathol..

[14] Perry R.N. (1996). Chemoreception in plant parasitic nematodes. Annu. Rev. Phytopathol..

[15] Curtis R.H.C. (2008). Plant-nematode interactions: Environmental signals detected by the nematode’s chemosensory organs control changes in the surface cuticle and behaviour. Parasite.

[16] Gang S.S., Hallem E.A. (2016). Mechanisms of host seeking by parasitic nematodes. Mol. Biochem. Parasitol..

[17] Rengarajan S., Hallem E.A. (2016). Olfactory circuits and behaviors of nematodes. Curr. Opin. Neurobiol..

[18] Tefft P.M., Bone L.W. (1985). Plant-induced hatching of eggs of the soybean cyst nematode, *Heterodera glycines*. J. Nematol..

[19] Rasmann S., Ali J.G., Helder J., van der Putten W.H. (2012). Ecology and evolution of soil nematode chemotaxis. J. Chem. Ecol..

[20] Pline M., Dusenbery D.B. (1986). Responses of plant-parasitic nematode *Meloidogyne incognita* to carbon dioxide determined by video camera-computer tracking. J. Chem. Ecol..

[21] Wang C., Bruening G., Williamson V.M. (2009). Determination of preferred pH for root-knot nematode aggregration using Pluronic F-127 gel. J. Chem. Ecol..

[22] Sommerville R.I., Davey K.G. (2002). Diapause in parasitic nematodes: A review. Can. J. Zool..

[23] Evans A.A.F., Perry R.N., Perry R.N., Moens M., Starr J.L. (2009). Survival mechanisms. Root-knot Nematodes.

[24] Lambert K.N., Bekal S. (2002). Introduction to plant parasitic nematodes. Plant Health Instr..

[25] Wyss U., Grundler F.M.W. (1992). Feeding behavior of sedentary plant parasitic nematodes. Eur. J. Plant Pathol..

[26] Sijmons P.C., Atkinson H.J., Wyss U. (1994). Parasitic strategies of root nematodes and associated host cell responses. Annu. Rev. Phytopathol..

[27] Bird D.M., Kaloshian I. (2003). Are roots special? Nematodes have their say. Physiol. Mol. Plant Pathol..

[28] Fuller V.L., Lilley C.J., Urwin P.E. (2008). Nematode Resistance. New Phytol..

[29] Stirling G.R., Pattison B. (2008). Beyond chemical dependency for managing plant-parasitic nematodes: Examples from the banana, pineapple and vegetable industries of tropical and subtropical Australia. Aust. Plant Pathol..

[30] Davies K.G., Spiegel Y., Jones J.T., Gheysen G., Fenoll C. (2011). Biological control of plant-parasitic nematodes: Towards understanding field variation through molecular mechanisms. Genomics and Molecular Genetics of Plant-Nematode Interactions.

[31] Williamson V.M., Kumar A. (2006). Nematode resistance in plants: The battle underground. Trends Genet..

[32] Petrussa E., Braidot E., Zancani M., Peresson C., Bertolini A., Patui S., Vianello A. (2013). Plant flavonoids-biosynthesis, transport and involvement in stress responses. Int. J. Mol. Sci..

[33] Winkel-Shirley B. (2001). Flavonoid biosynthesis: A colourful model for genetics, biochemistry, cell biology and biotechnology. Plant Physiol..

[34] Winkel-Shirley B. (2002). Biosynthesis of flavonoids and effects of stress. Curr. Opin. Plant Biol..

[35] Hassan S., Mathesius U. (2012). The role of flavonoids in root-rhizosphere signalling: Opportunities and challenges for improving plant-microbe interactions. J. Exper. Bot..

[36] Falcone Ferreyra M.L., Rius S.P., Casati P. (2012). Flavonoids: Biosynthesis, biological functions and biotechnological applications. Front. Plant Sci..

[37] Sugiyama A., Shitan N., Yazaki K. (2007). Involvement of a soybean ATP-binding cassette-type transporter in the secretion of genistein, a signal flavonoid in legume-*rhizobium* symbiosis. Plant Physiol..

[38] Badri D.V., Loyola-Vargas V.M., Broeckling C.D., De-la-Peña C., Jasinski M., Santelia D., Vivanco J.M. (2008). Altered profile of secondary metabolites in the root exudates of *Arabidopsis* ATP-binding cassette transporter mutants. Plant Physiol..

[39] Cesco S., Neumann G., Tomasi N., Pinton R., Weisskopf L. (2010). Release of plant-borne flavonoids into the rhizosphere and their role in plant nutrition. Plant Soil.

[40] Hawes M.C., Brigham L.A., Wen F., Woo H.H., Zhu Y. (2008). Function of root border cells in plant health: Pioneers in the rhizosphere. Annu. Rev. Phytopathol..

[41] Wuyts N., Swennen R., De Waele D. (2006). Effects of plant phenylpropanoid pathway products and selected terpenoids and alkaloids on the behaviour of the plant-parasitic nematodes *Radopholus similis*, *Pratylenchs penetrans* and *Meloidogyne incognita*. Nematology.

[42] Faizi S., Fayyaz S., Bano S., Yawar Iqbal E., Lubna, Siddiqi H., Naz A. (2011). Isolation of nematicidal compounds from *Tagetes patula* L. yellow flowers: Structure–activity relationship studies against cyst nematode *Heterodera zeae* infective stage larvae. J. Agric. Food Chem..

[43] Kampkötter A., Gombitang Nkwonkam C., Zurawski R.F., Timpel C., Chovolou Y., Wätjen W., Kahl R. (2007). Effects of the flavonoids kaempferol and fisetin on thermotolerance, oxidative stress and FoxO transcription factor DAF-16 in the model organism Caenorhabditis elegans. Arch. Toxicol..

[44] Kampkötter A., Timpel C., Zurawski R.F., Ruhl S., Chovolou Y., Proksch P., Wätjen W. (2008). Increase of stress resistance and lifespan of *Caenorhabditis elegans* by quercetin. Comp. Biochem. Physiol. Part B Biochem. Mol. Biol..

[45] Grünz G., Haas K., Soukup S., Klingenspor M., Kulling S.E., Daniel H., Spanier B. (2012). Structural features and bioavailability of four flavonoids and their implications for lifespan-extending and antioxidant actions in *C. elegans*. Mech. Ageing Dev..

[46] González J.A., Estévez-Braun A. (1998). Effect of (*E*)-Chalcone on Potato-Cyst Nematodes (*Globodera pallida* and *G. rostochiensis*). J. Agric. Food Chem..

[47] Cook R., Tiller S.A., Mizen K.A., Edwards R. (1995). Isoflavonoid metabolism in resistant and susceptible cultivars of white clover infected with the stem nematode *Ditylenchus dipsaci*. J. Plant Physiol..

[48] Vlachopoulos E.G., Smith L. (1993). Flavonoids in potato cyst nematodes. Fundam. Appl. Nematol..

[49] Goverse A., Smant G. (2014). The activation and suppression of plant innate immunity by parasitic nematodes. Annu. Rev. Phytopathol..

[50] Holbein J., Grundler F.M.W., Siddique S. (2016). Plant basal resistance to nematodes: An update. J. Exp. Bot..

[51] Zhao J., Dixon R.A. (2009). The ‘ins’ and ‘outs’ of flavonoid transport. Trends Plant Sci..

[52] Edwards R., Mizen T., Cook R. (1995). Isoflavonoid conjugate accumulation in the roots of lucerne (*Medicago sativa*) seedlings following infection by the stem nematode (*Ditylenchus dipsaci*). Nematologica.

[53] Dewick P.M. (1998). The biosynthesis of shikimate metabolites. Nat. Prod. Rep..

[54] Lambert K.N., Allen K.D., Sussex I.M. (1999). Cloning and characterization of an esophageal-gland specific chorismate mutase from the phytoparasitic nematode *Meloidogyne javanica*. Mol. Plant Microbe Interact..

[55] Jones J.T., Furlanetto C., Bakker E., Banks B., Blok V., Chen Q., Phillips M., Prior A. (2002). Characterization of a chorismate mutase from the potato cyst nematode *Globedera pallida*. Mol. Plant Pathol..

[56] Bekal S., Niblack T.L., Lambert K.N. (2003). A chorismate mutase from the soybean cyst nematode *Heterodera glycines* shows polymorphisms that correlate with virulence. Mol. Plant Microbe Interact..

[57] Doyle E.A., Lambert K.N. (2003). *Meloidogyne javanica* chorismate mutase 1 alters plant cell development. Mol. Plant Microbe Interact..

[58] Huang G., Dong R., Allen R., Davis E.L., Baum T.J., Hussey R.S. (2004). Two chorismate mutase genes from the root-knot nematode *Meloidogyne incognita*. Mol. Plant Pathol..

[59] Lambert K.N., Bekal S., Domier L.L., Niblack T.L., Noel G.R., Smyth C.A. (2005). Selection of *Heterodera glycines chorismate mutase-1* alleles on nematode-resistant soybean. Mol. Plant Microbe Interact..

[60] Vanholme B., Kast P., Haegeman A., Jacob J., Grunewald W., Gheysen G. (2009). Structural and functional investigation of a secreted chorismate mutase from the plant-parasitic nematode *Heterodera schachtii* in the context of related enzymes from diverse origins. Mol. Plant Pathol..

[61] Grundler F., Betka M., Wyss U. (1991). Influence of changes in the nurse cell system (syncytium) on sex determination and development of the cyst nematode *Heterodera schachtii*: Total amounts of proteins and amino acids. Physiol. Biochem..

[62] Jones J., Furlanetto C., Phillips M. (2007). The role of flavonoids produced in response to cyst nematode infection of *Arabidopsis thaliana*. Nematology.

[63] Wuyts N., Lognay G., Swennen R., De Waele D. (2006). Nematode infection and reproduction in transgenic and mutant *Arabidopsis* and tobacco with an altered phenylpropanoid metabolism. J. Exp. Bot..

[64] Huang J.-S., Barker K.R. (1991). Glyceollin I in soybean-cyst nematode interactions: Spatial and temporal distribution in roots of resistant and susceptible soybeans. Plant Physiol..

[65] Kaplan D.T., Keen N.T., Thomason I.J. (1980). Association of glyceollin with the incompatible response of soybean roots to *Meloidogyne incognita*. Physiol. Plant Pathol..

[66] Kaplan D.T., Keen N.T., Thomason I.J. (1980). Studies on the mode of action of glyceollin in soybean incompatibility to the root knot nematode, *Meloidogyne incognita*. Physiol. Plant Pathol..

[67] Abawi G.S., Van Etten H.D., Mai W.F. (1971). Phaseollin production induced by *Pratylenchus penetrans* in *Phaseolus vulgaris*. J. Nematol..

[68] Plowright R.A., Grayer R.J., Gill J.R., Rahman M.L., Harbornez J.B. (1996). The induction of phenolic compounds in rice after infection by the stem nematode *Ditylenchus angustus*. Nematologica.

[69] Baldridge G.D., O’Neill N.R., Samac D.A. (1998). Alfalfa (*Medicago sativa* L.) resistance to the root-lesion nematode, *Pratylenchus penetrans*: Defense-response gene mRNA and isoflavonoid phytoalexin levels in roots. Plant Mol. Biol..

[70] Soriano I.R., Asenstorfer R.E., Schmidt O., Riley I.T. (2004). Inducible flavone in oats (*Avena sativa*) is a novel defense against plant-parasitic nematodes. Phytopathology.

[71] Rich J.R., Keen N.T., Thomason I.J. (1977). Association of coumestans with the hypersensitivity of Lima bean roots to *Pratylenchus scribneri*. Physiol. Plant Pathol..

[72] Collingborn F.M.B., Gowen S.R., Mueller-Harvey I. (2000). Investigations into the biochemical basis for nematode resistance in roots of three *Musa* cultivars in response to *Radopholus similis* infection. J. Agric. Food Chem..

[73] Kennedy M.J., Niblack T.L., Krishnan H.B. (1999). Infection by *Heterodera glycines* elevates isoflavonoid production and influenes soybean nodulation. J. Nematol..

[74] Brueske C.H. (1980). Phenylalanine ammonia lyase activity in tomato roots infected and resistant to the root-knot nematode, *Meloidogyne incognita*. Physiol. Plant Pathol..

[75] Edens R.M., Anand S.C., Bolla R.I. (1995). Enzymes of the phenylpropanoid pathway in soybean infected with *Meloidogyne incognita* or *Heterodera glycines*. J. Nematol..

[76] Villeth G.R., Carmo L.S., Silva L.P., Fontes W., Grynberg P., Saraiva M., Mehta A. (2015). Cowpea–*Meloidogyne incognita* interaction: Root proteomic analysis during early stages of nematode infection. Proteomics.

[77] Klink V.P., Hosseini P., Matsye P.D., Alkharouf N.W., Matthews B.F. (2010). Syncytium gene expression in *Glycine max*[PI 88788] roots undergoing a resistant reaction to the parasitic nematode *Heterodera glycines*. Plant Physiol. Biochem..

[78] Shaukat S.S., Siddiqui I.A., Ali N.A., Ali S.A., Khan G.H. (2003). Nematicidal and allelopathic responses of *Lantana camara* root extract. Phytopathol. Mediterr..

[79] Oliveira J.T., Araujo-Filho J.H., Grangeiro T.B., Gondim D.M., Segalin J., Pinto P.M., Vasconcelos I.M. (2014). Enhanced synthesis of antioxidant enzymes, defense proteins and leghemoglobin in rhizobium-free cowpea roots after challenging with *Meloidogyne incognita*. Proteomes.

[80] Ithal N., Recknor J., Nettleton D., Hearne L., Maier T., Baum T.J., Mitchum M.G. (2007). Parallel genome-wide expression profiling of host and pathogen during soybean cyst nematode infection of soybean. Mol. Plant-Microbe Interact..

[81] Hutangura P., Mathesius U., Jones M.G.K., Rolfe B.G. (1999). Auxin induction is a trigger for root gall formation caused by root-knot nematodes in white clover and is associated with the activation of the flavonoid pathway. Funct. Plant Biol..

[82] Wasson A.P., Ramsay K., Jones M.G.K., Mathesius U. (2009). Differing requirements for flavonoids during the formation of lateral roots, nodules and root knot nematode galls in *Medicago truncatula*. New Phytol..

[83] Smant G., Helder J., Goverse A. (2018). Parallel adaptations and common host cell responses enabling feeding of obligate and facultative plant parasitic nematodes. Plant J..

[84] Kyndt T., Vieira P., Gheysen G., de Almeida-Engler J. (2013). Nematode feeding sites: Unique organs in plant roots. Planta.

[85] Bird A.F. (1961). The ultrastructure and histochemistry of a nematode-induced giant cell. J. Biophys. Biochem. Cytol..

[86] Palomares-Rius J.E., Escobar C., Vovlas A., Castillo P. (2017). Anatomical alterations in plant tissues induced by plant-parasitic nematodes. Front. Plant Sci..

[87] Cabrera J., Díaz-Manzano F.E., Barcala M., Arganda-Carreras I., de Almeida-Engler J., Engler G., Fenoll C., Escobar C. (2015). Phenotyping nematode feeding sites: Three-dimensional reconstruction and volumetric measurements of giant cells induced by root-knot nematodes in Arabidopsis. New Phytol..

[88] Davies L.J., Lilley C.J., Knox P., Urwin P.E. (2012). Syncytia formed by adult female *Heterodera schachtii* in *Arabidopsis thaliana* roots have a distinct cell wall molecular architecture. New Phytol..

[89] Peer W.A., Bandyopadhyay A., Blakeslee J.J., Makam S.N., Chen R.J., Masson P.H., Murphy A.S. (2004). Variation in expression and protein localization of the PIN family of auxin efflux facilitator proteins in flavonoid mutants with altered auxin transport in *Arabidopsis thaliana*. Plant Cell.

[90] Peer W.A., Murphy A.S. (2007). Flavonoids and auxin transport: Modulators or regulators?. Trends Plant Sci..

[91] Stenlid G. (1963). The effects of flavonoid compounds on oxidative phosphorylation and on the enzymatic destruction of indoleacetic acid. Physiol. Plant..

[92] Balasubramanian M., Rangaswami G. (1962). Presence of indole compound in nematode galls. Nature.

[93] Karczmarek A., Overmars H., Helder J., Goverse A. (2004). Feeding cell development by cyst and root-knot nematodes involves a similar early, local and transient activation of a specific auxin-inducible promoter element. Mol. Plant Pathol..

[94] Ng J., Perrine-Walker F., Wasson A., Mathesius U. (2015). The control of auxin transport in parasitic and symbiotic root–microbe interactions. Plants.

[95] Kyndt T., Goverse A., Haegeman A., Warmerdam S., Wanjau C., Jahani M., Engler G., de Almeida Engler J., Gheysen G. (2016). Redirection of auxin flow in Arabidopsis thaliana roots after infection by root-knot nematodes. J. Exp. Bot..

[96] Grunewald W., Cannoot B., Friml J., Gheysen G. (2009). Parasitic nematodes modulate PIN-mediated auxin transport to facilitate infection. PLoS Pathog..

[97] Jacobs M., Rubery P.H. (1988). Naturally occurring auxin transport regulators. Science.

[98] Yin R., Han K., Heller W., Albert A., Dobrev P.I., Zažímalová E., Schäffner A.R. (2014). Kaempferol 3-O-rhamnoside-7-O-rhamnoside is an endogenous flavonol inhibitor of polar auxin transport in *Arabidopsis* shoots. New Phytol..

[99] De Almeida Engler J., De Vleesschauwer V., Burssens S., Celenza J.L., Inzé D., Van Montagu M., Engler G., Gheysen G. (1999). Molecular markers and cell cycle inhibitors show the importance of cell cycle progression in nematode-induced galls and syncytia. Plant Cell.

[100] De Almeida Engler J., Gheysen G. (2012). Nematode-induced endoreduplication in plant host cells: Why and how?. Mol. Plant-Microbe Interact..

[101] Siddique S., Grundler F.M.W., Escobar C., Fenoll C. (2015). Metabolism in nematode feeding sites. Advances in Botanical Research.

[102] De Almeida Engler J., Kyndt T., Vieira P., Van Cappelle E., Boudolf V., Sanchez V., Gheysen G. (2012). CCS52 and DEL1 genes are key components of the endocycle in nematode-induced feeding sites. Plant J..

[103] De Almeida Engler J., Vieira P., Rodiuc N., Grossi de Sa M.F., Engler G., Escobar C., Fenoll C. (2015). The plant cell cycle machinery: Usurped and modulated by plant-parasitic nematodes. Advances in Botanical Research.

[104] Li Y., Duan S., Jia H., Bai C., Zhang L., Wang Z. (2014). Flavonoids from tartary buckwheat induce G2/M cell cycle arrest and apoptosis in human hepatoma HepG2 cells. Acta Biochim. Biophys. Sin..

[105] De Almeida Engler J., Rodiuc N., Smertenko A., Abad P. (2010). Plant actin cytoskeleton re-modeling by plant parasitic nematodes. Plant Signal. Behav..

[106] De Almeida Engler J., Van Poucke K., Karimi M., De Groodt R., Gheysen G., Engler G., Gheysen G. (2004). Dynamic cytoskeleton rearrangements in giant cells and syncytia of nematode-infected roots. Plant J..

[107] Böhl M., Tietze S., Sokoll A., Madathil S., Pfennig F., Apostolakis J., Fahmy K., Gutzeit H.O. (2007). Flavonoids affect actin functions in cytoplasm and nucleus. Biophys. J..

